# Technology-Facilitated Online Sexual Violence, Consent Negotiation, and Coping Among Adult Women: A Qualitative Study

**DOI:** 10.3390/healthcare14070863

**Published:** 2026-03-27

**Authors:** Azucena Martínez-Díaz, Pedro José López-Barranco, Ascensión Pilar Guillén-Martínez, Pedro Simón Cayuela-Fuentes, Gabriel Segura-López, Isabel María Pérez-Franco, César Leal-Costa, Ismael Jiménez-Ruiz

**Affiliations:** 1 Asociación Columbares, 30570 Beniaján, Spain; azucenamartinezdiaz@gmail.com; 2 Faculty of Social and Health Sciences, University of Murcia, 30800 Lorca, Spain; ascension.guillen@um.es; 3 Faculty of Nursing, University of Murcia, 30120 Murcia, Spain; pcayuela@um.es (P.S.C.-F.); gsegura@um.es (G.S.-L.); isabelmaria.perezf@um.es (I.M.P.-F.); 4 ICEBE: Research in Nursing Care and Evidence-Based Practice, Faculty of Nursing, University of Murcia, 30120 Murcia, Spain; 5 Advanced Nursing Care Research Group, Faculty of Nursing, University of Murcia, 30120 Murcia, Spain; ismael.jimenez@um.es

**Keywords:** social media, sexual violence, violence against women, consent, qualitative research

## Abstract

**Highlights:**

**What are the main findings?**

- Participants reported repeated exposure to diverse forms of online sexual violence (such as unsolicited sexual content, coercion, and persistent harassment), mostly perpetrated by strangers and often normalized or unrecognized as violence.
- These experiences undermine sexual consent, frequently conditioned by emotional manipulation, social pressure, or fear, and have significant psychological, emotional, and relational consequences.

**What are the implications of the main findings?**

- The findings underscore the need for comprehensive public policies, including early emotional and sexual education, social awareness campaigns, and improved institutional responses to digital sexual violence.
- Strengthening victim-centered support networks and regulating digital environments (through identity verification, platform accountability, and AI-based detection) may reduce impunity and enhance women’s safety online.

**Abstract:**

**Background/Objectives:** Online sexual violence is an increasingly prevalent form of gender-based harm facilitated by digital technologies, with significant consequences for the health, well-being, and rights of adult women. Despite growing attention to this phenomenon, women’s lived experiences remain underexplored, particularly regarding sexual consent and institutional responses. This study aimed to examine how adult women experience online sexual violence, how consent is negotiated or constrained in digital contexts, and how coping and institutional mechanisms are perceived. **Methods:** A qualitative study with a hermeneutic phenomenological approach was conducted. Data were collected through three focus groups with 23 women aged 21 to 42 years who were active users of social media. **Results:** Participants reported diverse forms of online sexual violence, including unsolicited sexual messages and images, persistent harassment, coercion, blackmail, and threats. Sexual consent was often undermined by emotional manipulation, social pressure, and fear, placing women in vulnerable positions. These experiences negatively affected well-being, contributing to anxiety, reduced self-esteem, fear, and difficulties in sexual and emotional relationships. Coping strategies were mainly individual, such as blocking perpetrators or reporting content, while social support was frequently perceived as insufficient. A generalized distrust of institutional responses emerged, with formal mechanisms viewed as ineffective or inaccessible. **Conclusions:** For the study participants, online sexual violence is increasingly normalized and concealed within digital environments, reinforced by anonymity and impunity. The findings highlight the need for continued research and the development of interventions that include early sexual and emotional education, awareness-raising initiatives, digital regulation, specialized professional training, and the strengthening of victim-centered support networks.

## 1. Introduction

The right of women to live free from violence is enshrined in various international agreements, including the 1979 Convention on the Elimination of All Forms of Discrimination against Women [1] and the United Nations Declaration on the Elimination of Violence against Women (1993) [2]. The declaration defines violence against women as ‘any act of gender-based violence that results in, or is likely to result in, physical, sexual or psychological harm or suffering to women, including threats of such acts, coercion or arbitrary deprivation of liberty, whether occurring in public or in private life’ [2].

According to global estimates by the World Health Organisation (WHO), one in three women has experienced physical and/or sexual violence at the hands of an intimate partner or non-partner at some point in their lives [3]. In Spain, the 2019 Macro-Survey on Violence against Women reported that 57.3% of women have experienced some form of gender-based violence, equivalent to 11.7 million women [4]. In 2024, Spanish courts received 199,084 reports of gender-based violence [5]. However, the United Nations (UN) notes that fewer than 40% of women affected by violence seek help and fewer than 10% submit an official complaint. These figures highlight the need to increase research and public awareness, and to improve measures to prevent gender-based violence [6].

In recent years, technological advances have given rise to new forms of violence. A 2024 study by the University of Murcia involving 1177 women found that 68.2% had experienced some form of online violence, with 62.7% specifically reporting online sexual violence [7]. These results are consistent with those of [8], who found that 83.6% of participants in their study had experienced at least one form of online violence in the previous 12 months.

Data from the National Statistics Institute (INE) indicate that, in 2024, 95.8% of the population aged 16 to 74 in Spain used the Internet, with instant messaging as the most common activity [9]. Meanwhile, the Spanish Data Protection Agency reports that digital violence against women and girls accounts for 70% of complaints received through the Priority Channel [10]. The UN defines this type of violence as ‘any act committed and spread through digital channels, such as social media, email, or mobile messaging apps, that harms dignity and integrity and hinders empowerment and the full enjoyment of human rights’ [11].

In 2022, the National Observatory of Technology and Society (ONTSI) published an alarming report on digital gender-based violence. More than half of women who had been harassed on social media reported experiencing panic attacks, anxiety, or stress. Forty-two per cent of victims of online harassment showed emotional stress, low self-esteem, and a loss of confidence [12]. These data underscore the need to clearly define and conceptualise digital violence in order to develop effective prevention and protection strategies. The United Nations Population Fund further emphasises that digital violence can lead to serious consequences, including stigmatisation, damage to reputation, reduced productivity, adverse effects on mental health, and social isolation [13]. The scientific literature provides further evidence supporting these findings. The study by [8] found that being a victim of online sexual violence is associated with lower psychological well-being.

To gain further insight into this issue, we conducted a qualitative study aimed primarily at exploring adult women’s perceptions and experiences in relation to online sexual violence, consent, and coping strategies.

## 2. Materials and Methods

### 2.1. Design

An exploratory qualitative study using a hermeneutic phenomenological design was conducted to explore and gain insight into the experiences of women who have been victims of gender-based violence through digital media, the limits of consent, and the coping strategies employed [14]. Within this framework, dialogue and co-construction of meaning allow access not only to individual accounts but also to relational dynamics that reveal tensions, negotiations, and nuances of experience. Accordingly, we used focus groups as a coherent dialogical device aligned with hermeneutic principles such as the part [15,16]. The paper has been prepared in accordance with the Consolidated Criteria for Reporting Qualitative Research (COREQ) [17].

### 2.2. Participants and Sample Size

The sample was selected using a combination of convenience sampling and snowball sampling. Participants were required to meet the following inclusion criteria: being female, over 18 years of age, and an active user of social media. The sole exclusion criterion was a lack of fluency in Spanish. The sample size was determined based on data saturation following the guidelines established by [18]. Theoretical saturation was defined as the recurrent emergence of codes across the focus groups. In the third group, no new codes related to the study aim emerged.

Participants were recruited through an anonymous survey in which they were presented with the main theme of the research. Women who were interested in participating contacted the principal investigator.

A total of 23 women participated in three focus groups. The first group comprised six women aged between 22 and 42. The second group comprised nine women aged between 21 and 28, and the third group comprised eight women aged between 21 and 39. View Table 1 for the characteristics of focus group participants (n = 23). None of the participants declined to take part.

### 2.3. Instrument and Study Variables

The instrument used was an ad hoc semi-structured script, designed for this study on the basis of a prior literature review.

The study examined the following dimensions: perceptions of the impact on overall health, self-esteem, and anxiety; coping strategies employed; barriers and facilitators to accessing support or resources; types of violence experienced (e.g., unsolicited messages, sextortion, etc.); the context of the violence (including the social media platform involved and the perpetrator’s identity); and consent.

These dimensions informed the design of an ad hoc semi-structured interview script, which covered the themes subsequently explored in the focus groups. These themes included experiences of online sexual violence; issues of consent and violence; the impact on health and well-being; coping strategies; and the needs, expectations, and recommendations of the participants.

### 2.4. Procedure and Facilitation of the Groups

Data collection was carried out by two researchers: (i) one with a doctoral degree (PhD) and experience in qualitative health research (male); and (ii) another, a pre-doctoral researcher previously trained in qualitative interviewing and health communication (female). Both received specific training to ensure consistency in the use of the semi-structured guide and in session facilitation; neither had any prior personal relationship with the participants.

The groups were conducted in person on university premises (45, 60, and 95 min), audio-/video-recorded, and transcribed verbatim. Moderation followed a reflexive, non-directive style aimed at fostering a safe environment, balancing speaking turns, preserving minority voices, normalizing disagreement, and avoiding leading prompts. Field notes were taken on interactional dynamics (e.g., affirmations/challenges, silences, overlaps) and were treated as data given their role in the group’s production of meaning.

In addition, the team acknowledged that their positioning and assumptions could influence both data generation and analysis. To address this, reflexive memos were written after each session and debriefings were held between the two interviewers to review facilitation decisions. This formed part of the investigator triangulation process, and the results were checked through participant feedback (member reflections/return).

### 2.5. Data Analysis

Data were analyzed using a hybrid inductive–deductive approach, consistent with Fereday and Muir-Cochrane [19]. This approach combined the emergent generation of codes and categories from participants’ narratives with the integration of relevant theoretical concepts. The process began with repeated, in-depth readings of the transcripts, from which an initial open coding was conducted.

Subsequently, initial codes were grouped into provisional categories through an iterative process of constant comparison. During this phase, a continuous comparison with the literature was undertaken as a flexible frame of reference, allowing the team to contextualize, refine, or nuance the emerging categories without imposing rigid external structures.

Credibility and interpretive coherence were strengthened through researcher triangulation, with coding conducted in pairs, working in parallel and then cross-checking results to reach consensus on analytic decisions. In addition, field notes were integrated to provide complementary information on group dynamics and to clarify interpretive nuances.

As part of validation procedures, member checking was carried out through shared reflections after each focus group, enabling participants to confirm preliminary interpretations, add nuance, and ensure that the findings faithfully represented their experiences.

The entire analytic process was managed with ATLAS.ti 25, which facilitated code organization, visualization of relationships between categories, and traceability of analytic decisions. To enhance transparency, a raw code list along with groundedness (frequency) values generated by ATLAS.ti 25 is provided in Appendix A Table A1 and Supplementary Materials Table S1.

Data analysis followed the inductive–deductive approach outlined by. The analysis began with thorough and repeated readings of the transcripts. The researchers then compared their interpretations to ensure consistency. Next, the main themes were identified, and the links between the categories were examined to understand their interrelationships. Credibility and reliability were reinforced by conducting the analysis in pairs using researcher triangulation. This approach ensured a systematic and collaborative process throughout all phases of the analysis. The researchers worked in parallel and then compared their observations. The qualitative data analysis software ATLAS.ti 25 was used to facilitate and organise the analytical process.

### 2.6. Ethical Considerations

This study received approval from the Ethics Committee of the University of Murcia (ACTA17/2025/CEI).

Participant data and information were processed in compliance with Organic Law 3/2018, of 5 December, on the Protection of Personal Data and Guarantee of Digital Rights (LOPDGDD) [20].

## 3. Results

### 3.1. Experiences of Online Sexual Violence

Participants described repeated exposure to different forms of online sexual violence occurring within their everyday use of social media platforms.

Across the three focus groups, women reported receiving unsolicited sexual messages and images, including explicit photographs and sexual propositions. These interactions were often described as frequent and sometimes recurrent over time.

*“They send you a photo of a penis, or some guy hits you up asking to be your sugar daddy…”*

(GF1)

*“Every week… I get offered money to have sex.”*

(GF2)

Participants also described persistent harassment, particularly when perpetrators created new accounts after being blocked in order to re-establish contact.

*“He created a new account to chat to me again… and then another one.”*

(GF1)

Some women reported situations involving emotional pressure, manipulation, or blackmail related to requests for intimate images. Others described being added to social media groups containing sexual content without their consent, particularly on Instagram.

*“On Instagram, especially.”*

(GF1)

*“They’ve even added me to Instagram groups…”*

(GF2)

Most participants indicated that these interactions were perpetrated by unknown individuals, often strangers encountered through social media platforms. In several cases, participants noted that the perpetrators appeared to be located in other countries. Violence perpetrated by acquaintances or partners was mentioned less frequently.

“They’ve all been strangers. Luckily, yes.”

(GF1)

“Guys they just knew by sight… would send them pics without being asked.”

(GF2)

Several participants also explained that they had initially interpreted these experiences as routine or expected aspects of online interaction, and only later began to reflect on them as forms of violence.

*“I didn’t think of it as sexual violence.”*

(GF2)

*“We’re getting used to that type of message.”*

(GF2)

### 3.2. Consent and Violence

Participants reflected extensively on how sexual consent was negotiated, interpreted, and sometimes constrained in both digital and interpersonal contexts. Their accounts revealed several situations in which consent was experienced as ambiguous, pressured, or conditioned, often influenced by misinformation, social expectations, and emotional dynamics.

#### 3.2.1. Online-Specific Dynamics Affecting Consent

Participants described situations occurring in digital interactions in which requests for intimate images or sexualised communication were accompanied by emotional pressure, persistence, or manipulation. This often involved tactics where perpetrators masked their intentions, creating a false sense of connection or offering incentives, which clouded the authenticity of any subsequent agreement.

*“As previously described, these matters are concealed and presented in a manner that renders them superficially benign or idyllic, when in fact they are not. Furthermore, the actors involved may be attempting to modify the participant’s thinking or to exert subtle coercion to secure compliance, including deception.”*

(GF3)

In some cases, this manipulation was linked to specific online phenomena, such as offers of money or a luxurious lifestyle, illustrating how digital platforms can facilitate new forms of conditioned consent.

*“For example, the phenomenon of ‘sugar daddies’ functions in this way: an older, wealthier individual approaches a younger person and offers to purchase whatever they desire. Essentially, this involves providing a lavish, almost godlike lifestyle in exchange for sexual relations.”*

(GF3)

Some women also referred to situations involving pressure to share intimate images, which they described as difficult to refuse in certain relational contexts. Participants also highlighted the ambiguity of digital communication, where written messages or online interactions could be interpreted in multiple ways, making it difficult to clearly express or interpret consent.

*“A message can be taken in many different ways.”*

(GF2)

#### 3.2.2. Interpersonal and Social Pressures Influencing Consent

Beyond digital interactions, participants also described broader interpersonal dynamics that shaped how they experienced and negotiated consent in intimate situations. These included emotional pressure, fear of conflict, concerns about rejection, and expectations within relationships. Emotional blackmail was a recurring theme, used to wear down resistance and create a sense of obligation.

*“Ultimately, emotional blackmail is employed, with statements such as ‘we have been together for a long time’ and ‘others do it and I do not’ used to exert pressure.”*

(GF3)

*“He used manipulation on me a lot. ‘He doesn’t love me’ or he would get angry. If I said no, he would get angry and stop talking to me.”*

(GF1)

Participants clearly identified that consent given under such circumstances was not authentic, describing it as a response to pressure rather than genuine desire.

*“In the end it is not real. You say yes but you do not want to. But they make you feel so bad that in the end you give in.”*

(GF3)

Some participants described situations in which they felt unable to interrupt or stop a sexual encounter despite feeling uncomfortable, highlighting the profound impact of fear and perceived power imbalances.

*“What was hard about stopping at that point was not a conscious fear, it was a very basic thing: here I am, alone in my house with someone stronger than me.”*

(GF1)

Social expectations related to sexuality also emerged as an important influence. Participants described feeling pressure to conform to certain experiences or behaviours considered appropriate for their age or social environment, a pressure that came not only from partners but also from peer groups.

*“It’s like we’re pigeonholed, expected to have done such and such by a certain age.”*

(GF2)

*“And not even from the man. But from your group of girlfriends. All the others do it and you don’t. You’re already too old.”*

(GF3)

In this context, several women reflected retrospectively on situations in which they had agreed to sexual encounters that they later understood as influenced by pressure or manipulation.

*“He made me skip school to go and have sex.”*

(GF1)

Participants also discussed misunderstandings and limited knowledge about consent, particularly during earlier stages of their lives. Some explained that they had only later begun to reinterpret certain experiences as forms of violence, having previously understood them as normal.

*“I didn’t realise it was sexual violence until I was older and talked to other people about it. I thought it was normal.”*

(GF1)

*“In my case no, I did not realize that it was sexual violence until I was older and I talked about it with other people. I thought that that was normal.”*

(GF1)

This delayed awareness was often linked to a broader societal normalization of certain behaviours, which made it difficult to identify coercion or violence at the time it occurred.

### 3.3. Impact on Health and Well-Being

Participants described experiencing psychological after-effects as a result of having been subjected to online sexual violence. These included anxiety, low self-esteem, fear, and profound disgust.

*‘I think it hits you from different angles: your self-esteem, your image…’*

(GF2)

*‘It disgusted me … it really disgusted me’*.

(GF2)

*‘It freaked me out … how can someone be so messed up?’*

(GF1)

Participants noted that the impact of online sexual violence is exacerbated when such acts are carried out in public and receive support or validation from others.

*‘It’s not just the people who call you a slut or say “this is for you”, but also those who back them up’*.

(GF1)

Moreover, this public exposure restricts their freedom to share content or interact on social media, for fear of being subjected to further violence or judgement.

*‘And so you go, well I won’t post a photo then, if that’s what they’re saying to me…’*

(GF2)

Participants felt especially fearful when they did not know the identity of the perpetrators. This is the case in most instances of online sexual violence.

*‘And then you think, how did this person even find my profile?’*

(GF2)

Finally, it is important to highlight the resignation and sense of helplessness expressed by the participants in reference to the normalisation of violence at both the individual and societal levels. They also voiced concerns about the development of artificial intelligence, highlighting its capacity to generate and disseminate sexualised imagery.

*‘We’re downplaying its importance when it’s actually a big deal’*.

(GF2)

In terms of the emotional and sexual consequences, participants emphasised that they felt disconnected from their own pleasure and found it difficult to trust other people. They explained that the need to constantly be on the lookout for potential attacks or uncomfortable situations prevents them from relaxing and enjoying their sexuality to the fullest. This constant vigilance directly interferes with their ability to experience pleasure and enjoyment: *‘I find it so much harder to enjoy myself’* (GF1).

In some cases, participants reported having had to dissociate to endure the situation: *‘I wasn’t really there, I just wanted it to be over, full-stop’* (GF1). In other instances, participants did not dissociate but instead denied the violence: *‘I just went along with it, end of story’* (GF1).

Once again, it is evident that many participants only realised they had been victims of sexual violence years after it occurred. Despite the impact these experiences had on their mental health and emotional well-being, their initial lack of awareness prevented participants from recognising their experiences as violence. This belated realisation often arose from conversations with other women or from participants’ growing understanding of consent and boundaries.

*‘I didn’t realise that what happened was wrong until many years later’*.

(GF1)

### 3.4. Coping Mechanisms

In terms of coping mechanisms, most participants reported taking individual measures, such as blocking accounts or reporting incidents on the platforms themselves. However, they never filed formal complaints.

*‘Block. Report on Instagram’.*

(GF2)

*‘It’s useless. You report it and nothing happens’*.

(GF1)

*‘You can resort to blocking, but it doesn’t matter, because you block them and they can just do it again’*.

(GF2)

While most participants took individual measures to address the violence they had experienced, such as blocking accounts or reporting incidents on social media platforms, they expressed deep distrust of institutional systems. Participants attributed this distrust to inadequate institutional responses and to the fear that professionals might not treat them with respect or credibility. They were concerned that professionals would judge, disbelieve, or ignore them, or minimise their experiences: *‘When it comes to social media, it doesn’t matter what you’re going to report, they’re just going to say no’* (GF1). This perception leads to a strong sense of helplessness, as participants feel that their efforts are not yielding any tangible results: *‘Reporting it is pointless’* (GF1).

With regard to the lack of an institutional response, participants emphasised key limiting beliefs that hinder formal reporting. These include the idea that a complaint cannot be filed without knowing the perpetrator’s identity and the assumption that solid evidence is necessary to proceed.

*‘You can’t file a complaint against anyone because you don’t know who they are’*.

(GF1)

In addition to personal strategies, social support emerged as a vital coping mechanism, with conversations among friends offering validation, awareness, and support. Participants emphasised the importance of breaking taboos and being able to openly discuss sex and sexual violence with their friends. Many noted that such conversations had been a turning point for them.

*‘It dawned on us as we were chatting amongst ourselves’*.

(GF1)

### 3.5. Perceived Needs and Expectations Emerging from Participants’ Experiences

Participants’ accounts revealed a series of perceived needs and expectations that emerged from their experiences of online sexual violence. These reflections were often framed in relation to the lack of education, support, and institutional responses they had encountered throughout their lives.

A recurrent theme across the focus groups was the perceived absence of comprehensive sexual and emotional education from an early age. Participants reflected on how this lack of education may have influenced their ability to recognise violence, understand consent, and set personal boundaries. They emphasised that discussions about sexuality, emotions, and respect were often limited or absent in their upbringing.

*‘Start from a very young age. In other spaces, at home, outside, from very early on. Using language that is tailored to their level and joyful’*.

(GF1)

Participants also described the importance of addressing emotional dimensions such as empathy, communication, and mutual respect in discussions about sexuality. Several women expressed that sexuality education had frequently been presented in a fragmented or gendered way, which they perceived as reinforcing misunderstandings around consent and relationships.

*‘And stress empathy and the emotional side, please. And a joint approach; in the end, it always seems like there is sex education for women and sex education for men’*.

(GF1)

Another issue raised during the discussions was the perceived lack of intergenerational dialogue about sexuality and digital risks. Participants described how conversations about sexual violence or online experiences were often absent within families, which contributed to feelings of isolation or uncertainty when confronting these situations.

*‘I think that besides educating kids from a really young age, right now we also need to educate parents. As all this happens because there’s a lack of education. Because those above us don’t have any. And teachers too’*.

(GF1)

*‘My parents’ generation doesn’t talk about this, nobody knows about it’*.

(GF1)

Participants also reflected on their experiences with institutional responses and highlighted the importance they attributed to professionals being adequately prepared to address situations of sexual violence. Their accounts suggested that fears of not being believed, judged, or taken seriously shaped their expectations regarding interactions with institutions.

*‘So they don’t treat you like a madwoman’*.

(GF1)

*‘These cases should be handled by police officers, solicitors, and judges with specific training’*.

(GF1)

In addition, participants frequently referred to characteristics of digital environments that, in their view, facilitated the persistence of online sexual violence. In this context, they discussed anonymity and the perceived lack of accountability on social media platforms.

*‘Zero anonymity. Name and ID number’*.

(GF1)

*‘AI should be used for this’*.

(GF1)

Finally, many participants emphasised the role of peer support and solidarity among women in helping them process and reinterpret their experiences. Conversations with friends and other women were described as important spaces for validation, awareness, and collective understanding of online sexual violence.

*‘There are a lot of support networks on social media’*.

(GF1)

Overall, these reflections illustrate how participants interpret their experiences and the gaps they perceive in education, social support, institutional responses, and digital environments.

## 4. Discussion

The aim of this study was to explore the experiences of adult women in relation to online sexual violence, the negotiation of consent, and their coping strategies. The findings confirm patterns identified in previous literature, but they also provide novel nuances regarding the normalization of abuse and the impact of digital environments.

Participants reported exposure to multiple forms of online sexual violence, including unsolicited sexual messages and images, persistent harassment, coercion, and blackmail. In line with previous studies, participants reported constant exposure to unsolicited sexual messages and images, mostly from strangers [8,21]. This is consistent with research documenting that up to 84% of young women have received sexually inappropriate online messages, often from unknown men [21]. Likewise, other studies report high prevalence rates (e.g., 66.7% of digital sexual harassment in the past 12 months) and note that 85% of women have witnessed, and 38% have directly experienced, online violence, reinforcing the population-level magnitude of the problem [8]. However, our study offers a novel perspective by identifying a process of “delayed awareness”: many participants did not initially recognise these behaviours as violence, but rather as a “normal” or “routine” part of social media use—an interpretation that aligns with research describing the early and everyday exposure to online harassment as a factor that promotes its normalisation and invisibility [21]. This normalisation of digital abuse represents a critical public health challenge, given its association with poorer psychological wellbeing (e.g., anxiety and depression) and even suicidal ideation or attempts among victims of online sexual harassment, as documented in population-based studies and recent analyses [8,22]. With respect to persistent harassment, where perpetrators create new accounts after being blocked by the victim, this behaviour falls under the term ‘technology-facilitated sexual violence’ (TFSV). This is characterised by persistent harassment facilitated by digital anonymity [23].

These findings align with recent theoretical and empirical work on technology-facilitated sexual violence, which highlights how digital anonymity, platform dynamics and sociocultural sexual scripts shape both online harm and the negotiation of consent [22]. Rather than confirming previous quantitative estimates, the present results illustrate established patterns, add contextual nuances that help explain their variability, and provide qualitative depth to mechanisms already described in the literature. This positioning clarifies how the experiences reported by participants fit within, refine or exemplify broader trends identified in contemporary research on TFSV.

With regard to consent, participants reported instances of conditioned or coerced consent resulting from emotional manipulation, blackmail, peer pressure, or fear. The literature confirms that many women consent without truly wanting to, and calls for a culture of consent in which respect for individual boundaries and assertiveness are at the forefront [24].

A prominent theme among participants was the presence of misinformation about consent and the assumption of an ‘obligation’ to adhere to a prescribed behavioural script. These findings align with those of [25], who emphasise the need to address sociocultural norms and sexual scripts in relation to consent. These authors advocate for a fundamental shift in approaches to teaching and understanding consent, framing it not only as a legal formality but also as a relational process influenced by social, cultural, and gender norms. They argue that this shift can be best achieved through critical education, personal empowerment, and effective communication [25].

With respect to the impact of online sexual violence on women’s health and well-being, participants described psychological consequences such as anxiety, low self-esteem, fear, and a profound sense of disgust. These findings are similar to those of other authors. These data are also consistent with those obtained by [26], whose study of adult women reported experiences of depression, anxiety, trauma, and body image dissatisfaction.

Participants described how experiences of online sexual violence negatively affected their emotional and sexual relationships. In their longitudinal study, Ref. [27] found that experiencing online sexual violence was significantly and negatively linked to the level of emotional and sexual intimacy in relationships, thereby undermining their quality.

Participants described various individual coping mechanisms, such as blocking accounts or reporting incidents on the platforms themselves. Ref. [28] found that account banning was generally rated highly and one of the most commonly used coping mechanisms among women.

The participants also emphasised the importance of social support as a coping mechanism: [29] found that the participants in their study sought support from family and friends, which helped them to recover emotionally, and [30] also highlighted the importance of social support as a primary coping mechanism for victims of online sexual violence, with support from family and the wider community being crucial for recovery.

Participants frequently expressed distrust toward institutional systems, as well as feelings of resignation and helplessness. These findings align with those of Watson’s scoping review, which revealed women’s accounts of inadequate support from the criminal justice system and their consequent distrust [31].

Participants’ reflections also revealed perceived gaps in education and social awareness that they associated with their experiences of online sexual violence. Many women described how the absence of comprehensive sexual and emotional education during childhood and adolescence influenced their ability to recognise certain behaviours as forms of violence or to interpret the dynamics of consent in digital interactions. Within their narratives, the call for earlier and more comprehensive education emerged not as a prescriptive recommendation, but as part of their retrospective interpretation of how their experiences could have been understood or addressed differently. Similarly, participants highlighted the perceived importance of greater social awareness regarding online sexual violence. Their accounts suggest that broader social recognition of these forms of violence could contribute to reducing their normalization and to fostering environments where women feel more supported in naming and confronting such experiences. These experiential reflections align with the findings of [32], which emphasize the crucial role of education in shaping understandings of sexual consent, sexual attitudes, and perceptions of social norms related to consent. From a phenomenological perspective, these insights illustrate how participants situate their personal experiences within wider social and educational contexts, interpreting the absence of dialogue, education, and awareness as factors that shaped their understanding of online sexual violence.

The fact that the study was conducted with Spanish-speaking women is also relevant. Cultural norms surrounding sexuality, communication, and gender relations in the Spanish context may shape how consent, violence, and interpersonal boundaries are understood and discussed. Participants’ accounts of limited intergenerational dialogue around sexuality reflect broader sociocultural patterns that may influence both the recognition of violence and help-seeking behaviors. In this sense, the findings should be interpreted within a Southern European cultural framework, where discussions around sexuality have historically been shaped by more conservative or private norms [23].

There is consensus among all participants on the need to regulate technology. In this vein, Ref. [33] suggest that technology could help to prevent sexual violence on social media by screening and controlling the content of online interactions.

### Limitations

This study has several limitations that should be considered when interpreting its findings. First, the sample size and sampling strategy limit the representativeness of the participants and therefore reduce the generalizability of the results to broader populations. Additionally, although focus groups are suitable for exploring shared experiences, group dynamics may influence disclosures, potentially reinforcing dominant narratives or discouraging participants from expressing more sensitive or divergent views. In addition, participation was voluntary and based on individual interest in the topic, which may have resulted in a self-selection bias. Women who chose to participate may have had prior experiences of online sexual violence or a greater willingness to reflect on and share these experiences.

Second, the analysis relies on self-reported and retrospective accounts, which may introduce recall bias or reinterpretation over time. This is particularly relevant in relation to consent, recognition of violence, and the delayed awareness reported by many participants. Moreover, the study includes only women who are active social media users, which excludes those with limited or no digital engagement and may restrict the diversity of experiences represented.

Finally, the qualitative design of the study means that findings should be understood as interpretive insights into the meanings and experiences constructed by the participants, rather than as estimates of prevalence or causal relationships. Despite these limitations, the study offers valuable perspectives on how online sexual violence is experienced, interpreted, and navigated by adult women.

## 5. Conclusions

This study provides a qualitative perspective on how adult women participating in the focus groups describe and experience online sexual violence, as well as how consent is negotiated in digital contexts. Participants reported experiences involving different forms of online sexual violence, often perpetrated by strangers, which in some cases may become normalized or are not initially recognized as violence. They also described situations in which consent was conditioned by emotional manipulation, social pressure, or fear, with potential repercussions for psychological well-being, self-esteem, and emotional and sexual relationships.

The coping strategies described were mainly individual, such as blocking perpetrators or reporting content on digital platforms, as well as seeking support from peers. However, participants also expressed distrust toward institutional responses and perceived limitations in formal reporting mechanisms.

The findings highlight the importance of strengthening educational, social, and institutional responses to online sexual violence, including comprehensive sexual and emotional education, awareness initiatives, specialized professional training, and the development of mechanisms that improve safety in digital platforms. Given the qualitative nature of the study and the limited sample size, these results should be interpreted as exploratory insights based on the experiences and perceptions of the participants.

Future research should explore these phenomena in more diverse populations, including individuals with different levels of digital engagement, as well as in different cultural contexts. Longitudinal and mixed-methods studies could further examine how experiences of online sexual violence evolve over time and how institutional responses can be improved. Additionally, research should focus on evaluating interventions aimed at prevention, education, and support for victims.

## Figures and Tables

**Table 1:** Characteristics of focus group participants.

Focal Group	Age	Education	Socio-Economic Level	Social Media Use (Hours/Day)
GF1	22	University studies	Low	1–3 h
GF1	24	University studies	Medium	1–3 h
GF1	27	University studies	Medium	3–5 h
GF1	31	University studies	Low	1–3 h
GF1	36	University studies	Medium	>5 h
GF1	42	University studies	High	3–5 h
GF2	21	University studies	Low	1–3 h
GF2	22	University studies	Low	<1 h
GF2	23	Secondary education	Low	1–3 h
GF2	24	University studies	Medium	1–3 h
GF2	25	Secondary education	Low	3–5 h
GF2	26	University studies	Medium	1–3 h
GF2	27	Secondary education	Medium	3–5 h
GF2	28	Secondary education	High	1–3 h
GF3	21	University studies	Low	1–3 h
GF3	24	University studies	Medium	>5 h
GF3	27	University studies	Low	1–3 h
GF3	30	University studies	Medium	3–5 h
GF3	33	University studies	Medium	1–3 h
GF3	36	University studies	Low	<1 h
GF3	39	University studies	Medium	3–5 h

## Data Availability

The data presented in this study are available on request from the corresponding author. Full interview transcripts are not publicly available due to ethical and confidentiality constraints; however, additional anonymized excerpts or redacted transcripts can be made available upon reasonable request to the corresponding author and subject to a data use agreement that ensures participant confidentiality.

## Supplementary Materials

The following supporting information can be downloaded at: https://www.mdpi.com/article/10.3390/healthcare14070863/s1, Table S1. Consolidated criteria for reporting qualitative studies (COREQ): 32-item checklist.

## Appendix A

**Table A1:** Code Groups, Groundedness (Frequency), and Relative Weight of Codes Identified in the Analysis.

Code Group	% of Group	Codes	% of Each Code
1. VSO Experiences	13.40%	⚬ Differences between VSO-VSP (13)	2.52%
		⚬ Online Sexual Experiences (6)	1.16%
		⚬ Online Sexual Experiences-Violence Identification (41)	7.95%
		⚬ Online sexual experiences-social network (4)	0.78%
		⚬ SELF-Perpetrator Experiences (5)	0.97%
2. Consent and violence	35.50%	⚬ Consent (78)	15.12%
		⚬ Consent definition (10)	1.94%
		⚬ Consent and Health Impact (5)	0.97%
		⚬ Non-Verbal Consent (10)	1.94%
		⚬ Verbal Consent (3)	0.58%
		⚬ Disinformation Consent (9)	1.74%
		⚬ Difficulties in setting limits (12)	2.33%
		⚬ Essentials of Consent (4)	0.78%
		⚬ Consent Influences (9)	1.74%
		⚬ Pressure & Consent (10)	1.94%
		⚬ Social Media & Consent (7)	1.36%
3. Impact on Health and Well-Being	6.80%	⚬ Affective-sexual consequences (2)	0.39%
		⚬ Dissociation (1)	0.19%
		⚬ Health Impact (32)	6.20%
4. Coping Mechanisms	27.50%	⚬ Individual Actions (14)	2.71%
		⚬ Coping-Distrust (12)	2.33%
		⚬ Coping & Resources (18)	3.49%
		⚬ Coping-non-reporting (13)	2.52%
		⚬ Social Support (4)	0.78%
		⚬ Evolution of violence (3)	0.58%
		⚬ Helplessness (10)	1.94%
		⚬ Institutional Intervention (4)	0.78%
		⚬ Structural Limitations (5)	0.97%
		⚬ Coping Mechanisms (61)	11.82%
		⚬ Normalization of violence (8)	1.55%
5. Needs and proposals	19.20%	⚬ Consent in campaigns (11)	2.13%
		⚬ Consent & Education (10)	1.94%
		⚬ Training for professionals (2)	0.39%
		⚬ Needs, expectations and proposals (40)	7.75%
		⚬ Perception of needs and supports (6)	1.16%
		⚬ Social Media (15)	2.91%
		⚬ Regulation Technology (12)	2.33%
		⚬ Social media and limits (10)	1.94%
		⚬ Sorority (1)	0.19%
		⚬ Suggestions (14)	2.71%
6. Social perceptions	7.60%	⚬ Gender Distancing (3)	0.58%
		⚬ Influence Pornography (5)	0.97%
		⚬ Social Perceptions (22)	4.26%
		⚬ Responsibility-Strength (2)	0.39%
		⚬ Responsibility-Behavior Modification (1)	0.19%
		⚬ Responsibility-Verbal Consent (1)	0.19%
		⚬ Women’s Responsibility (5)	0.97%
		⚬ Retrogression of feminism (6)	1.16%
		⚬ Taboo Sexuality (5)	0.97%

## References

[1] United Nations (1979). Convention on the Elimination of All Forms of Discrimination Against Women New York. https://www.ohchr.org/en/instruments-mechanisms/instruments/convention-elimination-all-forms-discrimination-against-women.

[2] United Nations Declaration on the Elimination of Violence Against Women. https://www.ohchr.org/en/instruments-mechanisms/instruments/declaration-elimination-violence-against-women.

[3] World Health Organization (2021). Violence Against Women Prevalence Estimates 2018.

[4] Delegación del Gobierno Contra la Violencia de Género (2020). Macroencuesta de Violencia Contra la Mujer 2019.

[5] Consejo General del Poder Judicial (2024). Informe Sobre Víctimas Mortales de La Violencia de Género y Doméstica En El Ámbito de La Pareja o Expareja En 2024.

[6] Unitd Nations The World’s Women 2015: Trends and Statistics. https://www.un.org/en/desa/world%E2%80%99s-women-2015-trends-and-statistics.

[7] Jiménez-Ruiz I., López-Barranco P.J., López-Yepes S., Suárez-Cortés M. Informe de Resultados: Investigación y Sensibilización Sobre Violencia contra las Mujeres en Redes Sociales 2025. https://columbares.org/wp-content/uploads/2024/03/Informe-de-resultados-de-investigacion-REDEX-2025.pdf.

[8] Martínez-Bacaicoa J., Henry N., Mateos-Pérez E., Gámez-Guadix M. (2024). Online Gendered Violence Victimization Among Adults: Prevalence, Predictors and Psychological Outcomes. Psicothema.

[9] Instituto Nacional de Estadística Población Que Usa Internet (En Los Últimos Tres Meses) Tipo de Actividades Realizadas Por Internet. https://www.ine.es/ss/Satellite?L=es_ES&c=INESeccion_C&cid=1259925528782&p=%5C&pagename=ProductosYServicios%2FPYSLayout&param1=PYSDetalle.

[10] Agencia Española de Protección de Datos La Violencia Digital Contra Mujeres y Niñas Aglutina el 70% de Los Casos Que Se Denuncian en el Canal Prioritario. https://www.aepd.es/prensa-y-comunicacion/notas-de-prensa/violencia-digital-contra-mujeres-y-ninas-aglutina-70-casos-canal-prioritario.

[11] Violencia Contra Mujeres y Niñas en el Espacio Digital: Lo Que es Virtual También es Real. https://mexico.unwomen.org/es/digiteca/publicaciones/2020-nuevo/diciembre-2020/violencia-digital.

[12] Observatorio Nacional de Tecnología y Sociedad (2022). Violencia Digital de Género: Una Realidad Invisible.

[13] United Nations Reporting Tip Sheet on Digital Violence: A Practical Reference Guide for Journalists and Media. https://www.unfpa.org/resources/digital-violence-tip-sheet-for-journalists.

[14] Mendieta-Izquierdo G., Ramírez-Rodríguez J.C., Fuerte J.A. (2015). La fenomenología desde la perspectiva hermenéutica de Heidegger: Una propuesta metodológica para la salud pública. Rev. Fac. Nac. Salud Pública.

[15] Bradbury-Jones C., Bradbury-Jones C., Sambrook S., Irvine F. (2009). The Phenomenological Focus Group: An Oxymoron. J. Adv. Nurs..

[16] Palmer M., Larkin M., de Visser R., Fadden G. (2010). Developing an Interpretative Phenomenological Approach to Focus Group Data. Qual. Res. Psychol..

[17] Tong A., Sainsbury P., Craig J. (2007). Consolidated Criteria for Reporting Qualitative Research (COREQ): A 32-Item Checklist for Interviews and Focus Groups. Int. J. Qual. Health Care.

[18] Malterud K., Siersma V.D., Guassora A.D. (2016). Sample Size in Qualitative Interview Studies: Guided by Information Power. Qual. Health Res..

[19] Fereday J., Muir-Cochrane E. (2006). Demonstrating Rigor Using Thematic Analysis: A Hybrid Approach of Inductive and Deductive Coding and Theme Development. Int. J. Qual. Methods.

[20] Boletín Oficial del Estado (2018). Ley Orgánica 3/2018, de 5 de Diciembre, de Protección de Datos Personales y Garantía de Los Derechos Digitales.

[21] Salerno-Ferraro A.C., Erentzen C., Schuller R.A. (2022). Young Women’s Experiences With Technology-Facilitated Sexual Violence From Male Strangers. J. Interpers. Violence.

[22] Benítez-Hidalgo V., Henares-Montiel J., Ruiz-Pérez I., Pastor-Moreno G. (2024). Cyber Sexual Harassment against Women and Impact on Health. A Cross-Sectional Study in a Representative Population Sample. J. Public Health.

[23] Powell A., Henry N. (2019). Technology-Facilitated Sexual Violence Victimization: Results From an Online Survey of Australian Adults. J. Interpers. Violence.

[24] Gomez-Pulido E., Garrido-Macías M., Miss-Ascencio C., Expósito F. (2024). Under the Shadows of Gender Violence: An Exploration of Sexual Consent through Spanish University Women’s Experiences. Eur. J. Psychol. Appl. Leg. Context.

[25] Williamson L., Bayly M., Poncelet E., Lawson K. (2023). A Qualitative Exploration of Undergraduate Student Perspectives of Sexual Consent within a Sexual Script Framework. Can. J. Hum. Sex..

[26] Iroegbu M., O’Brien F., Muñoz L.C., Parsons G. (2024). Investigating the Psychological Impact of Cyber-Sexual Harassment. J. Interpers. Violence.

[27] Stockman D., Uzieblo K., Fomenko E., Littleton H., Keygnaert I., Lemmens G., Verhofstadt L. (2024). Psychological Distress and Relational Intimacy Following Sexual Violence: A Longitudinal Study. Psychol. Belg..

[28] Schoenebeck S., Lampe C., Triệu P. (2023). Online Harassment: Assessing Harms and Remedies. Soc. Media Soc..

[29] Pijlman V., Boertien E. (2025). A Comparative Study of the Help-Seeking Behavior of Victims of Contact Sexual Violence and Image-Based Sexual Harassment and Abuse. Violence Women.

[30] Sheikh M.M.R., Rogers M.M. (2024). Technology-Facilitated Sexual Violence and Abuse in Low and Middle-Income Countries: A Scoping Review. Trauma Violence Abus..

[31] Watson S. (2024). Online Abuse of Women: An Interdisciplinary Scoping Review of the Literature. Fem. Media Stud..

[32] MacDougall A., Craig S., Goldsmith K., Byers E.S. (2022). Sexual Consent Attitudes and Behaviour: Associations with Sexual Health Education, Sexual Consent Education, and Sexual Attitudes. Can. J. Hum. Sex..

[33] Wei M., Consolvo S., Kelley P.G., Kohno T., Matthews T., Meiklejohn S., Roesner F., Shelby R., Thomas K., Umbach R. (2024). Understanding Help-Seeking and Help-Giving on Social Media for Image-Based Sexual Abuse. arXiv.

