# Supplementary material for: Technology-Facilitated Online Sexual Violence, Consent Negotiation, and Coping Among Adult Women: A Qualitative Study
Source: Healthcare (Basel). 2026 Mar 27;14(7):863. doi: 10.3390/healthcare14070863 (PMC13073261; doi:10.3390/healthcare14070863)
Supplement: Supplementary file 1 [file healthcare-14-00863-s001.zip › healthcare-4165169-supplementary.pdf]

Supplementary Table S1. Consolidated criteria for reporting qualitative studies (COREQ): 32-item checklist

No. Item	Guide questions/description	Reported on Page #
**Domain 1: Research team and reflexivity**		
Personal Characteristics		
1. Inter viewer/facilitator	Data collection was carried out by two researchers: (i) one with a PhD and experience in qualitative health research; and (ii) another, a pre-doctoral researcher previously trained in qualitative interviews and health communication.	Section 2.4. Procedure and facilitation of the groups
2. Credentials	one with a PhD and experience in qualitative health research; and (ii) another, a pre-doctoral researcher previously trained in qualitative interviews and health communication.	Section 2.4. Procedure and facilitation of the groups
3. Occupation	The professional role is described: researcher with experience in qualitative health research and a pre-doctoral researcher trained in qualitative interviews and health communication.	Section 2.4. Procedure and facilitation of the groups
4. Gender	Was the researcher male or female?	Section 2.4. Procedure and facilitation of the groups
5. Experience and training	Specific training and experience are reported: the PhD researcher has experience in qualitative health research; the predoctoral researcher was previously trained in qualitative interviews and health communication; in addition, both received specific training to ensure consistency.	Section 2.4 Procedure and facilitation of groups, first paragraph and second paragraph.
Relationship with participants		
6. Relationship established	There was no prior personal relationship between the interviewers and the participants.	Section 2.4 Procedure and facilitation of groups, first paragraph
7. Participant knowledge of the interviewer	It is noted that the participants contacted the principal investigator following an anonymous survey in which they were presented with the main topic of the research.	Recruitment described in 2.2 Participants and sample size
8. Interviewer characteristics	The manuscript acknowledges the team's positioning and describes reflective measures: reflective memos after each session, debriefing sessions among interviewers, triangulation of researchers, and verification by members. It indicates that field notes were taken and member	Section 2.4 Group procedure and facilitation, paragraphs on field notes and reflexivity; also in 2.5 Data

	feedback was used.	analysis (triangulation, member verification).
**Domain 2: study design**		
Theoretical framework		
9. Methodological orientation and Theory	A phenomenological hermeneutic design and the use of hermeneutic principles are declared; preparation according to COREQ is also mentioned.	Section 2.1 Design, first paragraph.
Participant selection		
10. Sampling	Convenience sampling and snowball sampling were used.	Section 2.2 Participants and sample size, first paragraph
11. Method of approach	Recruitment via anonymous survey; interested parties contacted the principal investigator.	Section 2.2 Participants and sample size, paragraph on recruitment
12. Sample size	23 women in three focus groups (6, 9 and 8 participants).	Section 2.2 Participants and sample size, paragraph with figures and Table 1.
13. Non-participation	How many people refused to participate or dropped out? Reasons?	Section 2.2 Participants and sample size, paragraph on recruitment
Setting		
14. Setting of data collection	Where was the data collected? e.g. home, clinic, workplace.	Section 2.4 Procedure and facilitation of the groups, first paragraph.
15. Presence of nonparticipants	Was anyone else present besides the participants and researchers? No se informa si hubo observadores, personal de apoyo o acompañantes presentes durante los grupos.	Section 2.4 Procedure and facilitation of the groups, first paragraph.
16. Description of sample	Age, educational level, socioeconomic status, and social media use (hours/day) are provided in Table 1; inclusion/exclusion criteria are also indicated.	Section 2.2 Participants and sample size and Table 1.
Data collection		
17. Interview guide	It is indicated that a semi-structured ad hoc script designed based on a literature review was used, and the dimensions/topics explored are listed.	Section 2.3 Instrument and study variables.

18. Repeat interviews	Were repeat inter views carried out? If yes, how many?	No repeated interviews were conducted.
19. Audio/visual recording	It is noted that the sessions were recorded in audio/video format and transcribed verbatim.	Section 2.4 Procedure and facilitation of the groups, second paragraph
20. Field notes	Field notes were taken on interactional dynamics and integrated as data.	Section 2.4 Procedure and facilitation of the groups, second paragraph; also referenced in 2.5 Data analysis.
21. Duration	The durations reported are: 45, 60, and 95 minutes for the three groups.	Section 2.4 Procedure and facilitation of the groups, second paragraph.
22. Data saturation	It is indicated that the sample size was determined by saturation; theoretical saturation was defined and reached in the third group (no new codes emerged).	Sección 2.2 Participants and sample size, párrafo sobre saturación.
23. Transcripts returned	Member checking is mentioned through shared reflections after each focus group (verification by members).	Section 2.5 Data analysis, paragraph on validation.
**Domain 3: analysis and findings**<br>*Data analysis*		
24. Number of data coders	Coding was performed in pairs; therefore, there were two coders.	Location: Methods, section 2.5 Data analysis (add).
25. Description of the coding tree	Did authors provide a description of the coding tree?	Sección 2.5 Data analysis. Appendix 1.
26. Derivation of themes	The manuscript declares a hybrid inductive-deductive approach (Fereday & Muir-Cochrane), whereby themes were derived through a combination of emergent generation and comparison with theoretical frameworks.	Section 2.5 Data analysis, first paragraph.
27. Software	The use of ATLAS.ti 25 to manage the analytical process is explicitly indicated.	Section 2.5 Data analysis, last paragraph.
28. Participant checking	It is reported that member checking was carried out through shared reflections after each focus group (member feedback) to confirm preliminary interpretations.	Section 2.5 Data analysis, paragraph on validation
Reporting		

29. Quotations presented	Quotations illustrating the topics are included.	Location: Results 3.1 Experiences of online sexual violence.
30. Data and findings consistent	There is methodological consistency: percentages of coded segments are reported, illustrative quotations are presented, and triangulation and verification procedures are described. However, the absence of a coding tree and the need for greater traceability of quotations limit external evaluation.	Location: Methods 2.5 and Results 3.1–3.2.
31. Clarity of major themes	The main topics are clearly identified and quantified in Results. Experiences of online sexual violence (13.4%) y Consent and violence (35.5%),	Location: Results
32. Clarity of minor themes	Secondary topics and variations are mentioned (e.g., unknown vs. known perpetrators; international location of aggressors; initial normalisation of messages; inclusion in Instagram groups; emotional coercion/sextortion), but they are not formalised as a subsection.	Location: Results
